# New CACNA1A deletions are associated to migraine phenotypes

**DOI:** 10.1186/s10194-018-0891-x

**Published:** 2018-08-30

**Authors:** G. S. Grieco, S. Gagliardi, I. Ricca, O. Pansarasa, M. Neri, F. Gualandi, G. Nappi, A. Ferlini, C. Cereda

**Affiliations:** 1 IRCCS Mondino Foundation, Genomic and post-Genomic Center, Pavia, Italy; 2 Unit of Medical Genetics, S. Anna University-Hospital, Ferrara, Italy; 3 IRCCS Mondino Foundation, Headache Science Center, Pavia, Italy

**Keywords:** *CACNA1A*, Deletion, Migraine phenotypes, De novo

## Abstract

**Background:**

Familial hemiplegic migraine type 1 (FHM1) is a form of migraine with aura caused by heterozygous mutations in 4 genes: *CACNA1A, ATP1A2, SNC1A* and *PRRT2*, but further heterogeneity is expected. Here have been described clinical and molecular features in patients suffering from migraine with Aura (MA), without (MO) and hemiplegic migraine attacks.

Next Generation Sequencing by TruSeq Custom Amplicon for *CACNA1A* and *ATP1A2* gene has been performed. All genetic variants have been confirmed by Sanger sequencing and all samples were also analyzed with MLPA assay for *ATP1A2-CACNA1A* genes to detect duplication or deletion. All MLPA data were verified by Real Time PCR.

**Results:**

Sequencing analysis showed 3 point mutations, two novel variants and one already described in literature. Moreover, MLPA analysis showed 3 deletions in 9 sporadic hemiplegic migraine (18%), in 3 patients with non-hemiplegic migraine (4.1%) and in 3 patients affected by episodic ataxia (20%). Two sporadic patients showed a deletion in exons 41–43, while the rest of HM patients (5) showed a deletion in the terminal part of the *CACNA1A* gene.

About episodic ataxia, we have identified deletions in exon 12–15 and in exon 47. Finally, in migraine patients, we have found different subjects affected by different phenotypes deleted in exon 47.

**Conclusion:**

This work highlights the importance to complement analysis as direct sequencing with quantitative analysis (MLPA). In fact, intragenic *CACNA1A* rearrangements have been detected. Our work demonstrated that deletions in *CACNA1A* gene may be associated also to different migraine phenotypes.

## Background

*CACNA1A* gene encodes the α1 subunit of neuronal Ca_V_2.1 (P/Q-type) voltage-gated calcium channels that are widely expressed throughout the central nervous system (CNS). Mutations in the *CACNA1A* gene have been found to be responsible for three disorders with autosomal dominant inheritance: i) Episodic Ataxia 2 (EA2; MIM: 108500), ii) familial hemiplegic migraine type 1 (FHM1; MIM: 141500), and iii) spinocerebellar ataxia type 6 (SCA6; MIM: 183086). Clinical overlap between the three disorders in terms of symptoms has been previously reported [1–3]. Interesting, Pradotto and collaborators [4], described, for the first time, a common mutation in *CACNA1A* gene in different phenotypes, such as EA2 and SCA6, opening a new prospective about the association between distinct phenotypes caused by the same *CACNA1A* mutation.

About FHM1, more than 20 missense mutations are known to be associated with a broad spectrum of clinical features besides hemiplegic migraine [5]. Mutations in *CACNA1A* gene explain around 30–50% of the FHM cases [5], in fact, other genes as *ATP1A2, SCN1A, PRRT2* have been associate to FHM1 disease [5–7].

So far, there is no evidence for works investigating deletions or duplication in *CACNA1A* in patients FHM1 while in EA2 patients, different deletions [8–10] in *CACNA1A* gene have been found to lead to loss-of-function of recombinant human CaV2.1 channels. About the last CACNA1A associated disorder, SCA6 disease, *CACNA1A* caused the pathology by the expansion of CAG repeat in the α1A subunit of the voltage-dependent calcium channel gene [11].

Moreover, a relevant number of EA2 and FHM cases remain genetically undiagnosed, it can be imputed to incomplete sequencing analysis of the *CACNA1A* gene, to genetic heterogeneity [12–15] or to the occurrence of CNVs undetectable by exon sequencing. Indeed, in recent years, quantitative analyses as MLPA or QMPSF allowed to identify large genomic deletions involving the *CACNA1A* gene and associate to EA2 and different *CACNA1A* related phenotypes.

This work would emphasize the importance of the genetic rearrangement of *CACNA1A* gene in different phenotype and also to highlight the importance of using multiple techniques, in order to reach a higher diagnostic sensitivity and specificity.

Herein, we present an important screening of *CACNA1A* gene to identify new point mutations (by Next Generation Sequencing) and to search CNVs (using MLPA assay) in patients affected by hemiplegic migraine, episodic ataxia and, for the first time, in migraine with aura not hemiplegic (MA) and migraine without aura (MO). To perform a complete analysis of *CACNA1A* gene, we have also investigated the expansion of the CAG repeat in the α1A subunit of *CACNA1A* associated to SCA6 phenotype.

## Methods

### Patients and controls

The clinical diagnosis followed the IHS 2nd edition criteria having collected a written informed consent to genetic tests at “C. Mondino” Neurological Institute in Pavia and at the University-Hospital S. Anna in Ferrara.

We collected peripheral blood samples of 50 patients with hemiplegic migraine (36 sporadic and 14 familial cases), of 15 patients with Episodic Ataxia2 (EA2) and of 72 patients with MA or MO. Moreover we enrolled 70 healthy volunteers age 65 years or more after obtaining written informed consent (Table 1).

**Table 1:** Baseline Characteristics

HM		migraine		EA2	CTR
SPORADIC	FAMILIAR	MA	MO		
36	14	36	36	15	70

### Sequencing analysis

We purified genomic DNA from blood by automatic extraction (Maxwell® 16 Blood DNA – Promega). Next Generation Sequencing by TruSeq Custom Amplicon for CACNA1A, ATP1A2 SCN1A, PRRT2 genes has been performed (Illumina) according to the manufacturers’ instructions. DNA analysis has been carried out using Illumina MySeq. All genetic variants have been confirmed by Sanger sequencing, PCR-amplified the 47 exons and 80 base pairs of the flanking intronic sequences of *CACNA1A* (NC_000019.9). All patients have been also screened for ATP1A2, SCN1A, PRRT2 mutations.

### MLPA and real time PCR

Search of multi exon rearrangements was performed by MLPA assay using the P348-A2 ATP1A2-CACNA1A probe mix (SALSA MLPA Kit, MRC-Holland). Identification of deletion/duplications was confirmed by quantitative real –time PCR (qPCR). PCR reactions were carried out using a Biorad IQ5 (BioRad) in a 96-well optical plate with a final reaction volume of 50 μl. About 10 ng of DNA were subjected to thermal cycling conditions with a pre-run of 2 min at 50 °C and 10 min at 95 °C. Cycle conditions were 40 cycles at 95 °C for 15 s and 60 °C for 1 min according to the Sybr Green Real Time PCR Protocol (primers are available upon request). Analysis of relative gene expression data have been performed using real-time quantitative PCR and the 2–ΔΔCT method [16], the starting copy number of the unknown samples was determined in comparison with the known copy number of the calibrator sample, using the 2–(ΔΔCt) method. As a reference gene, we used *GAPDH*. *P* Values in patients were compared to results obtained in 70 normal individuals, deemed free of neurological disorders. All the experiments have been performed in triplicates.

### Microsatellite STR analysis

All patients and healthy controls have been genotyped for the (CAG)n microsatellite markers. Accordingly to the already published method [17], all samples have been run by sequencer DNA analyser ABI 3031 (microsatellite analysis) and analysed by the Gene Mapper software.

## Results

### Sequencing analysis

**Fig. 1:**
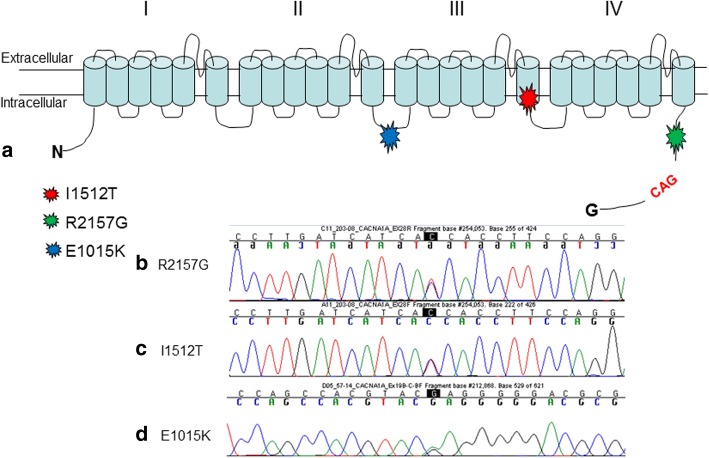
Newly identified and previously reported *CACNA1A* deletions

Genetic analysis of the *CACNA1A* gene with direct sequencing, in a cohort of migraine sufferers (HM and MA/MO) identified 3 different point mutations (Table 2; Fig. 1a).

**Table 2:** List of detected point mutations and phenotypical features

Patients	Mutation	Present age (yrs)	Age of onset (years)	Migraine/headache Features	Frequency	Duration (h)	Cerebellar sign and symptoms	Other clinical features
57/14	E1015K	53	31	Hemiplegic migraine	na	variable	no cerebellar	no
263/12	I1512T	63	28	Familiar Hemiplegic migraine	1–2/week	variable	no cerebellar	no
203/08	I1512T	46	18	Familiar Hemiplegic migraine	1–2/week	variable	no cerebellar	no
209/13	R2157G	39	21	Familiar Hemiplegic migraine	na	variable	no cerebellar	no

The first patient affected by FHM showed the missense mutation E1015K, already described in literature [18] as pathogenetic.

Next, we have identified two new mutations, not reported in literature or in disease databases. The first one is the missense variation Arg2157Gly (R2157G) that we found in a Moroccan hemiplegic migraine patient; unfortunately no relatives are available for segregation analysis (Fig. 1b). In silico analysis with Polyphen software (http://genetics.bwh.harvard.edu/pph2/) predicted the R2157G mutation as probably damaging with a score of 0.998. The pathogenity has been confirmed also by SIFT and MutationTaster software (http://sift.jcvi.org/ and http://www.mutationtaster.org/).

The second novel missense mutation, I1512T, has been detected in heterozygosis in two brothers with FHM.

This variation has an amino acidic substitution corresponding to an Isoleucine into a Threonine, (I1512T) two essential amino acids that have a chiral side chain.

In silico analysis with Polyphen software predicted the I1512T mutation as probably damaging with a score of 0.999. These predictions were confirmed using Predict Protein (https://www.predictprotein.org).

### MLPA results

The samples analysed showed the presence of an intragenic deletion in 9 subjects with sporadic hemiplegic migraine (18%), in 3 patients with non-hemiplegic migraine (4.1%) and in 3 patients affected by episodic ataxia (20%) (Table 3).

**Table 3:** Clinical Features

Patients	Mutation	Present age (yrs)	Age of onset (years)	Migraine/headache Features	Frequency	Duration (h)	Cerebellar sign and symptoms	Other clinical features
16/11	del 41–43	17	14	Hemiplegic migraine	5/year	12–48	No cerebellar	No
16/13	del 41–44	42	12	Hemiplegic migraine	2/month	1–2	No cerebellar	Painful paresthesias and mild cognitive deficit
124/11	del 37–47	4	6 (months)	Hemiplegic migraine	2/month	2–3	No cerebellar	Nystagmus, emiplegia, distonia
72/12	del 45–47	19	15	Hemiplegic migraine	6/year	48–72	No cerebellar	Mild cognitive deficit
117/14	del 46–47	39	33	Hemiplegic migraine	2/month	variable	No cerebellar	Muscle pain and fatigue, drop head, loss of deambulation
12/15	del 47	42	23	Hemiplegic migraine	10/year	variable	No cerebellar	No
27/14	del 47	16	20	Hemiplegic migraine	12/year	48–72 h	No cerebellar	No
118/14	del 47	23	17	Hemiplegic migraine	2–3/month	2–3 h	No cerebellar	No
75/13	del 47	13	9	Hemiplegic migraine	2–3/month	variable	No cerebellar	No
187/12	del 12–15	44	12	Episodic ataxia (familiar)	1–2/month	2–3 h	Nystagmus in horizontal and vertical gaze and mild cerebellar signs	Monolateral neurosensory hearing loss
106/16	del 12–15	17	15	Episodic ataxia (familiar)	1–2/week	variable	Multidirectional gaze evoked nystagmus, dysdiadochokinesis, mild difficulty in tandem walking	Congenital stationary night blindness
165/14	del 47	6	1	Episodic ataxia	1/month	variable	No cerebellar	No
195/12	del 47	14	7	MO	2/month	variable	No cerebellar	No
237/12	del 47	60	8	MA	6/year	variable (10 min to 48–72 h)	No cerebellar	Multifocal leukoencephalopathy
80/11	del 47	38	9	MO	2–3/month	variable	No cerebellar	No

Sporadic hemiplegic migraine: two patients showed a deletion in exons 41–43, while the rest of HM patients [5] showed a deletion in the terminal part of the *CACNA1A* gene (ex 47).

Episodic ataxia: in a female EA patient (and her affected son) we identified a deletion in exon 12–15, while a second patient showed a deletion in exon 47.

Migraine: the three deleted patients showed the same deletion in exon 47, but two different phenotypes, as MA [1] and MO [2].

Also in one healthy control we have detected a deletion in exon 47.

### Microsatellites

Expansion of the CAG repeat in the α1A subunit of the voltage-dependent calcium channel gene CACNA1A, leads to the Spinocerebellar ataxia type 6 (SCA6, MIM 183086). This is a late-onset, slowly progressive neurodegenerative disorder accounting for between 6 and 32% of families with autosomal dominant ataxia [11]. None of the patients had expanded CAG repeats in the 3’-UTR of *CACNA1A*.

## Discussion

Here we report an exhaustive screening of the CACNA1A gene in a large sample of 137 patients with four clinical phenotypes: FHM1, sporadic HM, EA2, MA and MO.

The study was addressed through Next Generation Sequencing and MLPA analyses in the patients.

In the last years an increasing number of point mutations in CACNA1A have been reported as causative of FHM-1 and EA2 [19, 20].

In our cohort of patients, Sanger analysis identified only three point mutations in the CACNA1A gene; the E1015K, previously described associated with different migraine phenotypes [18], was identified in a patient with a clinical diagnosis of Hemiplegic migraine without family history.

Two novels variations (I1521T, R2157G) have been detected in three unrelated patients with FHM and in silico studies predicted both the variations as damaging.

Patients with EA2 and MA/MO phenotypes resulted negative to sequencing analysis, suggesting that other genes, known to be causative of FHM such as ATP1A2, SNC1A and PRRT2, could be involved and are worth to be screened. Our results expand the number of small mutations of CACNA1A gene associated with HM/FHM phenotype but on the other hand highlight the need to carry out a second level analysis to search for gross genomic rearrangement.

Therefore, in our cohort of patients negative to Sanger sequencing, we performed MLPA analysis of CACNA1A gene and we found different deletions in the C-terminus region of the gene.

To date, deletions in the CACNA1A gene have been reported in literature but associated exclusively to an EA2 phenotype.

Riant and collaborators [8] described a deletion spanning from exon 38 to 40 and removing 105 amino acids in the proximal half of the cytoplasmatic C-terminal tail of the protein, that is essential for channel function. This result has been reported also by others [9, 10].

We identified a novel deletion of exons 12–15 in a female patient with EA2 and in her son affected by congenital night blindness. The deletion of 12–15 exons is out of frame and predicts to cause premature truncation of the CACNA1A protein product; or its complete absence via mRNA decay.

The novelty of this report is the intrafamilial phenotypic variability associated to the same deletion, giving rise to a classical EA2 in the mother and to a more complex phenotype in the son.

**Fig. 2:**
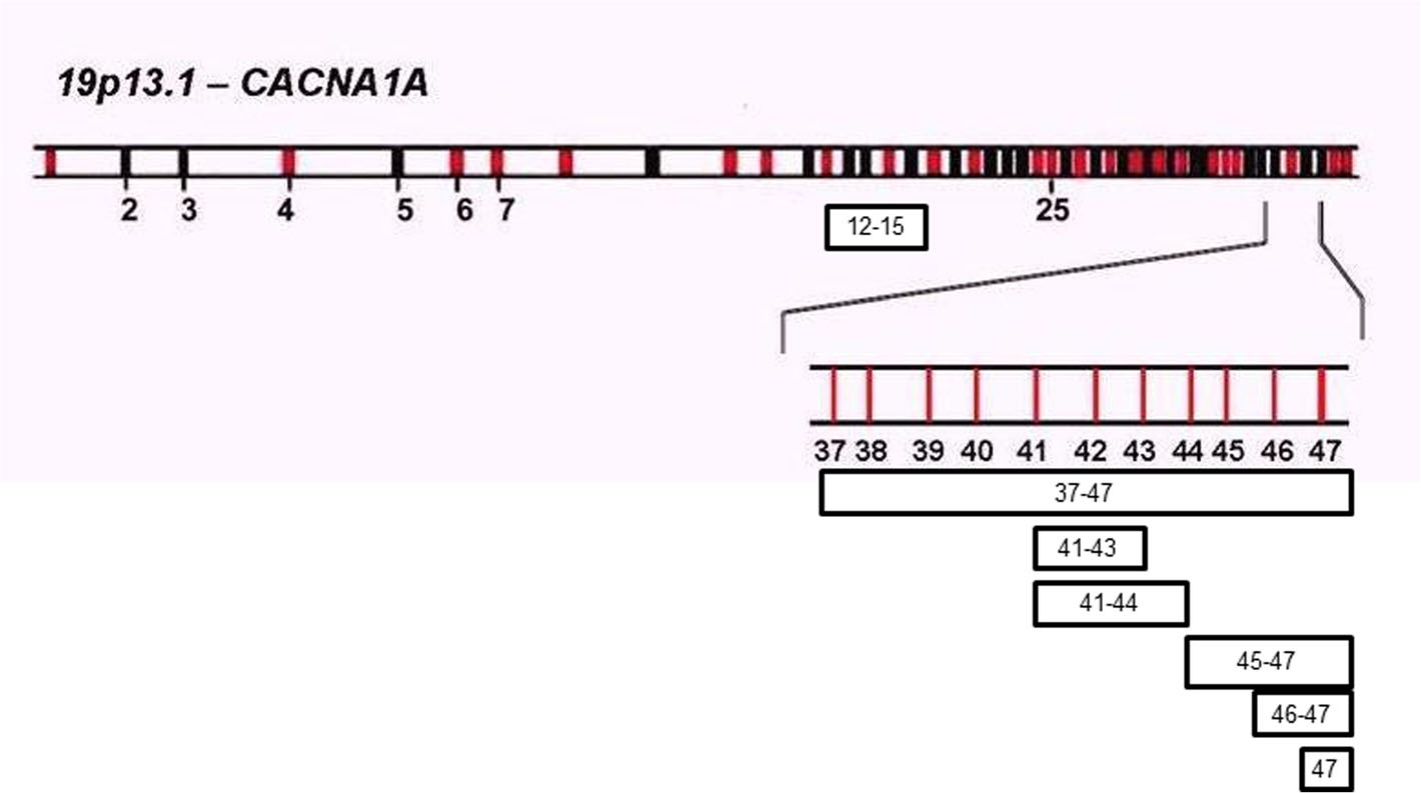
CACNA1A deletions

A part from this family, in patients with migraine (HM, MA and MO), we have detected different deletions in the 3′ end of CACNA1A (Fig. 2).

Deletions of more than one exon were present only in patients with sporadic HM, instead the single exon 47 deletion was found associated to different clinical presentations: HM (four cases), EA2 (one case), MO (two cases) and MA (one case).

The high frequency of exon 47 deletion in our cohort lead us to speculate if it might be a polymorphism or a mutational hot spot, harbouring also the triplet repeat involved in the pathogenesis of SCA6.

We have detected the single exon 47 deletion in one subject of the controls cohort and in this subject, clinical investigation was negative for neurological involvement and he did not refer migraine; we cannot exclude a low penetrance of the deletion in this patient or that he will manifest clinical signs in the years to come.Further investigation and functional studies are necessary to better understand the role of CACNA1A exon 47 deletion in migraine disorders.

Our findings expand the spectrum of clinical phenotype associated with deletions in the 3′ end of CACNA1A gene. Interestingly, all our deleted patients manifested early motor and headache symptoms in the juvenile period or even in preteen years. If this correlate to the specific function of the gene product during development is unclear, although mutations in other FHM genes (ATP1A2 and SCN1A) may occur frequently in episodic ictal brain dysfunctions in childhood. The other reports of deletions in CACNA1A gene are in keeping with these findings, suggesting that deletions in the gene are associated with an onset of clinical manifestations before the age of 30 [8, 9].

## Conclusions

In conclusion, the four clinical phenotypes we have included in this study (FHM1, sporadic HM, EA2, MA and MO) should be considered part of the same disease spectrum and for this group of diseases it should be kept in mind the genetic and allelic heterogeneity and also the intrafamilial / interfamilial clinical variability.

Moreover, in patients with migraine phenotypes and episodic ataxia is becoming mandatory the screening of gross rearrangements of CACNA1A gene for a complete genetic workup.

## Abbreviations

CACNA1A: Calcium voltage-gated channel subunit alpha1 A
FHM: Familiar Hemiplegic Migraine
MA: Migraine with Aura
MO: Migraine without Aura
